# Sulfated and non-sulfated chondroitin affect the composition and metabolism of human colonic microbiota simulated in an in vitro fermentation system

**DOI:** 10.1038/s41598-023-38849-5

**Published:** 2023-07-29

**Authors:** Kentaro Inokuma, Daisuke Sasaki, Kaoru Kurata, Megumi Ichikawa, Yuya Otsuka, Akihiko Kondo

**Affiliations:** 1 Graduate School of Science, Technology and Innovation, Kobe University, 1-1 Rokkodai-cho, Nada-ku, Kobe, 657-8501 Japan; 2 Glycoscience, Central Research Laboratory, Seikagaku Corporation, 3-1253, Tateno, Higashiyamato, Tokyo 207-0021 Japan; 3 Biomass Engineering Program, RIKEN, 1-7-22 Suehiro-Cho, Tsurumi-ku, Yokohama, Kanagawa 230-0045 Japan

**Keywords:** Bacteria, Metagenomics, Microbiology, Gastrointestinal models

## Abstract

Chondroitin sulfate (CS) is a family of glycosaminoglycans and have a wide range of applications in dietary supplements and pharmaceutical drugs. In this study, we evaluated the effects of several types of CS, differing in their sulfated positions, on the human colonic microbiota and their metabolites. CS (CSA, CSC, and CSE) and non-sulfated chondroitin (CH) were added into an in vitro human colonic microbiota model with fecal samples from 10 healthy individuals. CS addition showed a tendency to increase the relative abundance of *Bacteroides*, *Eubacterium*, and *Faecalibacterium*, and CSC and CSE addition significantly increased the total number of eubacteria in the culture of the Kobe University Human Intestinal Microbiota Model. CSE addition also resulted in a significant increase in short-chain fatty acid (SCFA) levels. Furthermore, addition with CSC and CSE increased the levels of a wide range of metabolites including lysine, ornithine, and Ile-Pro-Pro, which could have beneficial effects on the host. However, significant increases in the total number of eubacteria, relative abundance of *Bacteroides*, and SCFA levels were also observed after addition with CH, and the trends in the effects of CH addition on metabolite concentrations were identical to those of CSC and CSE addition. These results provide novel insight into the contribution of the colonic microbiota to the beneficial effects of dietary CS.

## Introduction

Glycosaminoglycans (GAGs) are linear polysaccharides comprising repeating disaccharide units of amino sugars (*N*-acetylglucosamine [GlcNAc] or *N*-acetylgalactosamine [GalNAc]) and either hexuronic acid or hexose that are universally present in all animals, including humans, as major components of the extracellular matrix. GAGs can be classified into several types according to their disaccharide units and the linkages between sugars. Chondroitin sulfate (CS) is a representative family of GAGs that are ubiquitously present on cell surfaces and within extracellular matrices. CS comprises disaccharide units alternating β (1–4)-linked GalNAc and β (1–3)-linked glucuronic acid (GlcUA) bearing sulfate groups at various positions and has been identified based on its characteristic disaccharide composition (Supplementary Fig. S1). CSA is predominantly composed of an A-unit in which the C-4 position of GalNAc is sulfated. CSC mainly comprises a C-unit with a sulfate group at the C-6 position of GalNAc. CSE has a composition rich in E-units in which the C-4 and C-6 positions of GalNAc are sulfated. Non-sulfated chondroitin (CH) comprises only a non-sulfated unit (O-unit).

CS is particularly known as a major component of articular cartilage and has been implicated in chondrocyte proliferation and differentiation^1^. It has demonstrated therapeutic immunomodulatory and anti-inflammatory effects^2^ and is used as a dietary supplement and pharmaceutical drug for the treatment of joint diseases, including osteoarthritis^3^. Moreover, CS has been recently reported involved in the regulation of various physiological events, such as organogenesis, cytokinesis, morphogenesis, and central nervous system development^4, 5^. Several studies have shown that oral administration of CS reduces the incidence of coronary events in patients with coronary heart disease^6–8^, and Melgar-Lesmes et al.^9^ suggested that these cardioprotective effects of CS may arise from modulation of proinflammatory activation of endothelium and monocytes and foam cell formation.

Despite its broad physiological roles and wide range of applications, the mechanisms underlying the beneficial effects of dietary CS remain unclear. CS is a high-molecular-weight polysaccharide that is poorly absorbed by the body when administered orally^10, 11^. However, some intestinal bacteria can degrade and utilize CS for their growth^12, 13^. Shang et al.^14^ reported that dietary CS and its derivatives altered the composition of colonic bacteria in mice. Since the gut microbiota exerts a wide effect on host physiology, the beneficial effects of oral administration of CS on the host are considered to be due to the modulation of colonic microbiota and their metabolites. However, ethical considerations have constrained human interventional clinical trials, and the functionality of orally administered CS in humans remains unclear. In particular, the effects of CS sulfation patterns on the colonic microbiota and their metabolites are largely unknown.

In this study, we investigated the possible effects of various CSs, including CH, on human colonic microbiota using an in vitro human colonic microbiota model, the Kobe University Human Intestinal Microbiota Model (KUHIMM)^15, 16^. KUHIMM is a single-batch anaerobic fermentation system for the metagenomic and metabolic simulation of human colonic microbiota. Fecal samples from 10 healthy individuals were individually cultivated in KUHIMM with or without each CS, and the total eubacterial growth, microbial composition, and metabolites after cultivation were analyzed to evaluate the effects of these CSs on human colonic microbiota.

## Results

### Consumption of CSs by human colonic microbiota in KUHIMM

Fecal samples from 10 Japanese volunteers were individually cultivated anaerobically in KUHIMM with each CS (CH, CSA, CSC, and CSE) at 37 °C for 48 h. The concentrations of each type of CS in the culture broth at 0 and 48 h are shown in Supplementary Table S1, and the residual ratio of CSs after 48 h of incubation are summarized in Table 1. In most cultivation experiments, the residual ratio of CSs was low (< 20%), indicating that most of the added CSs were degraded or absorbed by the human colonic microbiota simulated by the KUHIMM and consumed. In contrast, no remarkable consumption of CSA was observed in the cultivation with fecal sample HS-02 (Supplementary Table S1), suggesting failure of cultivation. Therefore, the culture sample of the CSA/HS-02 combination was excluded from further analysis.

**Table 1:** Residual ratio of CSs after 48 h of incubation in KUHIMM.

Fecal samples	Residual ratio (%)			
	CH	CSA	CSC	CSE
HS-01	2.8	4.1	4.2	8.9
HS-02	1.7	–^a^	1.3	1.4
HS-03	1.5	1.5	1.4	1.8
HS-04	1.4	1.7	1.5	1.8
HS-05	1.3	1.3	1.2	1.5
HS-06	1.2	1.3	1.3	1.8
HS-07	1.7	1.5	1.5	17.0
HS-08	1.2	1.3	1.0	1.5
HS-09	1.5	1.4	1.4	2.0
HS-10	2.6	1.6	9.2	1.9
Mean ± SD	1.7 ± 0.6	1.7 ± 0.9	2.4 ± 2.6	4.0 ± 5.1

^a^No significant CSA consumption was observed during cultivation.

### Effects of CS addition on structure of human colonic microbiota in KUHIMM

**Figure 1:**
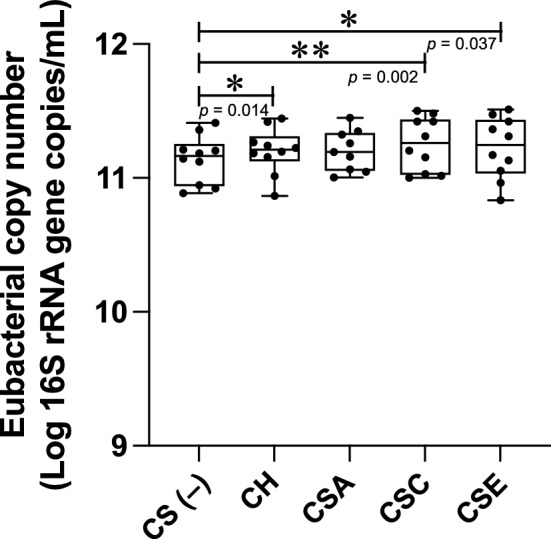
Eubacterial copy number in KUHIMM after 48 h of cultivation with each CS (CH, CSA, CSC, or CSE) or without CS (CS(–)). Data are shown as the median and interquartile range (25th–75th percentiles) of 10 samples (9 for CSA only). *p < 0.05, **p < 0.01. Wilcoxon matched-pairs signed-rank test.

The microbial composition of original fecal samples and that of samples that cultivated in KUHIMM with or without each CS for 48 h were analyzed by sequencing, covering the V3–V4 hypervariable regions of the bacterial 16S rRNA gene. KUHIMM without CS addition was used as the control. An average of 128,649 high-quality leads was obtained for each sample (Supplementary Table S2). The bacterial operational taxonomic unit numbers and Chao1 value for species richness were lower in the CS non-added KUHIMM culture than in the original fecal samples (p = 0.029 and 0.052, for operational taxonomic unit numbers and Chao1, respectively; Mann–Whitney *U*-test, Supplementary Table S2). However, there was no significant difference in the count of operational taxonomic unit between the CS-added and non-added KUHIMM cultures (CH: *p* = 0.388; CSA: *p* = 0.127; CSC: *p* = 0.325; CSE: *p* = 0.211; Mann–Whitney *U*-test, Supplementary Table S2). The Shannon index for species diversity was lower in the CS non-added KUHIMM culture group than in the original fecal sample group (*p* = 0.002; Mann–Whitney *U*-test, Supplementary Table S2). However, there was no significant difference in the values between the CS-added and non-added KUHIMM cultures (CH: *p* = 0.905; CSA: *p* = 0.651; CSC: *p* = 0.912; CSE: *p* = 0.604; Mann–Whitney *U*-test, Supplementary Table S2). The Simpson index for species diversity was lower in the CS non-added KUHIMM culture group than in the original fecal sample group (*p* = 0.003; Mann–Whitney *U*-test, Supplementary Table S2); however, no significant difference was found in the values between the CS-added and non-added KUHIMM cultures (CH: *p* = 0.968; CSA: *p* = 0.739; CSC: *p* > 0.999; CSE: *p* = 0.661; Mann–Whitney *U*-test, Supplementary Table S2). These results confirmed that the diversity of the colonic microbiota did not change upon the addition of 0.3% CSs in KUHIMM. On the other hand, CH, CSC, and CSE addition significantly increased the number of total eubacteria in KUHIMM compared to the control culture (CH: *p* = 0.014; CSC: *p* = 0.002; CSE: *p* = 0.037; Wilcoxon matched-pairs signed-rank test, Fig. 1).

**Figure 2:**
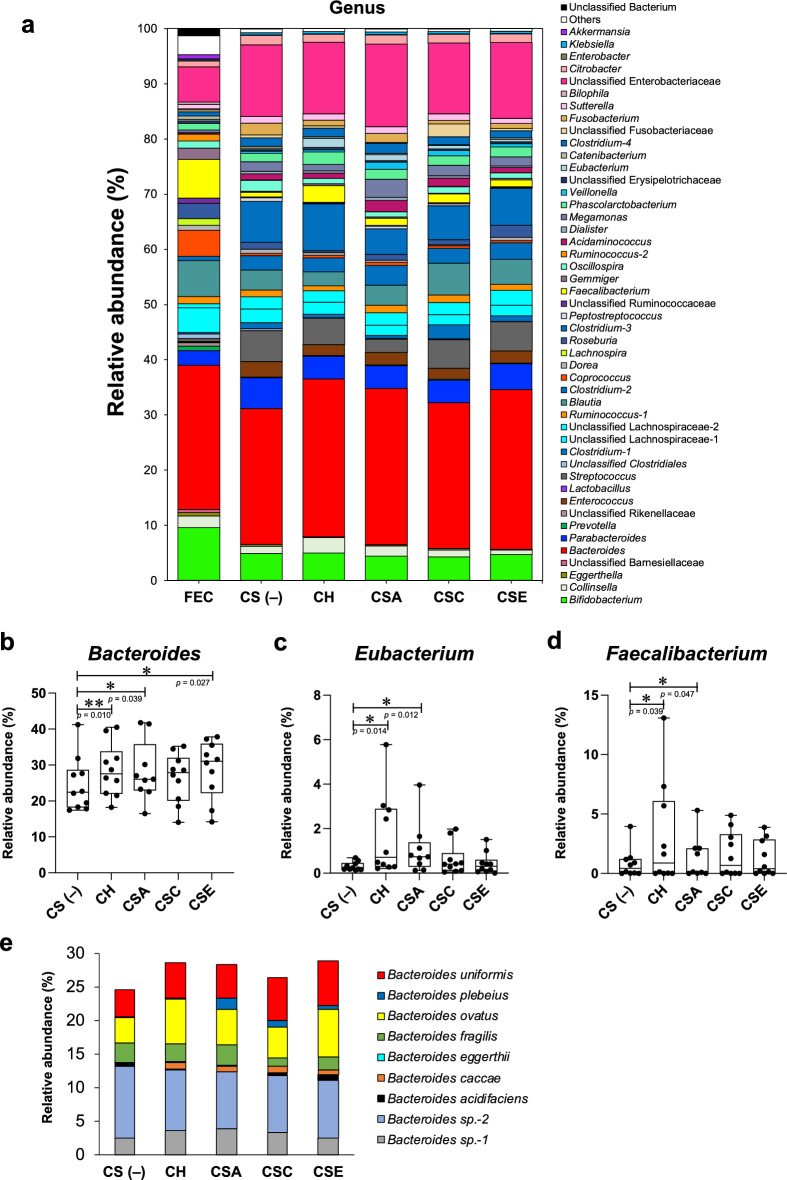
Effect of 0.3% CS addition on the relative abundance of the microbiota of each sample. (**a**) Genus-level compositional view of bacteria in original feces (FEC) and KUHIMM after 48 h of cultivation with each CS (CH, CSA, CSC, or CSE) or without CS (CS(–)). Data are shown as the average relative abundances in 10 samples (9 for CSA only). Genera with lower abundance (< 1.0%) and lower levels of similarity (< 99%) were indicated as Others and Unclassified bacterium, respectively. (**b**–**d**) The relative abundance of *Bacteroides* (**b**), *Eubacterium* (**c**), and *Faecalibacterium* (**d**) in KUHIMM. Data are shown as the median and interquartile range (25th–75th percentiles) of 10 samples (9 for CSA only). *p < 0.05, **p < 0.01. Wilcoxon matched-pairs signed-rank test. (**e**) The relative abundance of *Bacteroides* species in KUHIMM. Data are shown as the average relative abundances in 10 samples (9 for CSA only).

Figure 2a presents the relative abundance of the microbiota of each sample at the genus level. The relative abundance of *Bacteroides* in the KUHIMM cultures tended to increase with CS addition (Fig. 2a) and was significantly increased by CH, CSA, and CSE addition in comparison to the control culture (CH: *p* = 0.010; CSA: *p* = 0.039; CSE: *p* = 0.027; Wilcoxon matched-pairs signed-rank test, Fig. 2b). The relative abundances of *Eubacterium* (CH: *p* = 0.014; CSA: *p* = 0.012; Wilcoxon matched-pairs signed-rank test, Fig. 2c) and *Faecalibacterium* (CH: *p* = 0.039; CSA: *p* = 0.047; Wilcoxon matched-pairs signed-rank test, Fig. 2d), which belong to the phylum Firmicutes, were significantly increased by CH and CSA addition. Changes in the relative abundances of these genera in the KUHIMM inoculated with each fecal sample are shown in Supplementary Fig. S2. For the other genera, no significant increases in the relative abundance were observed between the CS-added and non-added KUHIMM cultures.

While CS addition increased the relative abundance of *Bacteroides* at the genus level, the breakdown of this increase at the species level differed depending on the CS addition (Fig. 2e). The relative abundance of *Bacteroides ovatus* and *Bacteroides caccae* increased after addition with any type of CS, whereas that of *Bacteroides plebeius* increased only after addition with CSA, CSC, and CSE, but not CH.

### Effect of CS addition on short-chain fatty acid (SCFA) production

**Figure 3:**
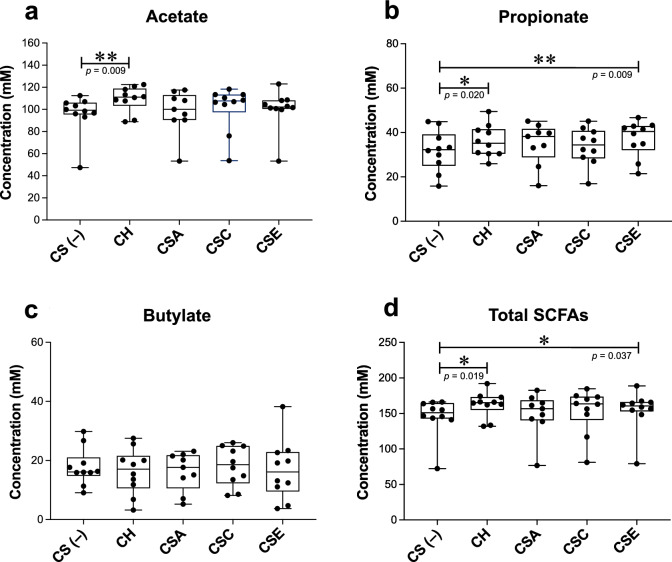
Concentration of SCFAs after 48 h of cultivation in KUHIMM with each CS (CH, CSA, CSC, or CSE) or without CS (CS(–)). Data are shown as the median and interquartile range (25th–75th percentiles) of 10 samples (9 for CSA only). *p < 0.05, **p < 0.01. Wilcoxon matched-pairs signed-rank test.

To evaluate the effect of CSs on the production of SCFAs, which play an essential role in maintaining human health, the levels of three typical SCFAs, namely acetate, propionate, and butyrate, were measured after 48 h of cultivation in KUHIMM with or without CS (Fig. 3). The acetate level was significantly increased by CH addition (*p* = 0.009; Wilcoxon matched-pairs signed-rank test, Fig. 3a). Meanwhile, the propionate level was significantly increased by CH and CSE addition (*p* = 0.020 and *p* = 0.009, respectively; Wilcoxon matched-pairs signed-rank test, Fig. 3b). However, butyrate levels were not significantly altered by addition with any type of CS (Fig. 3c). Changes in the levels of SCFAs in the KUHIMM inoculated with each fecal sample are shown in Supplementary Fig. S2.

### Comprehensive quantification of metabolites

To further investigate the effect of CH, CSC, and CSE on bacterial metabolism in the human colon, metabolites in the supernatant of KUHIMM cultures were comprehensively quantified. After 48 h of cultivation in KUHIMM, 348 metabolites were detected in the supernatant, of which 75 showed significant concentration changes after CS addition (Supplementary Table S3). Table 2 lists all metabolites for which a significant > twofold change in concentration was observed after each CS addition. Most of these metabolites increased in concentration after CS addition. Among them, the concentrations of the basic amino acids lysine and ornithine were particularly increased by addition with CH (12.7- and 3.8-fold, respectively, compared to non-addition) and CSE (8.8- and 7.1-fold, respectively, compared to non-addition). However, only 3-(4-hydroxyphenyl)propionic acid, 2-(4-hydroxyphenyl)propionic acid, or 3-(3-hydroxyphenyl)propionic acid significantly decreased in concentration by less than half after CS addition.

**Table 2:** Comprehensive relative quantification of metabolites.

Compound	Ratio vs CS (–)^a^		
	CH	CSC	CSE
Adenine	3.0*	2.4*	2.4*
GalNAc or GlcNAc	2.3*	1.7	2.0*
*O*-Acetylhomoserine or 2-aminoadipic acid	2.4*	1.8	2.1*
*N*-Acetylmethionine	1.4	2.7*	1.5
*m*-Ethoxybenzoic acid, 3-phenyllactic acid, or *p*-methoxyphenylacetic acid	0.9	3.9*	1.1
Glycerol 3-phosphate	5.3*	1.9*	2.2*
Homoserine	3.0*	2.4*	2.8*
2-Hydroxy-4-methylvaleric acid	1.1	2.3*	1.2
3-(4-Hydroxyphenyl)propionic acid, 2-(4-hydroxyphenyl)propionic acid, or 3-(3-hydroxyphenyl)propionic acid	0.4	0.4*	0.5*
Ile-Pro-Pro	3.7	4.1*	3.9*
Lysine	12.7*	5.5	8.8*
Ornithine	3.8*	1.5	7.1*
Saccharopine	2.1*	2.3*	2.0
*N*^2^-Succinylornithine	3.2*	3.0	1.1
Ala-Ala or XC0145	2.0*	1.2	1.5*

^a^The values are shown as a fold-change in the average concentration of each metabolite in 10 samples relative to that detected in the KUHIMM after 48 h of cultivation without CSs (CS (–)). *p < 0.05, Wilcoxon signed-rank test.

## Discussion

CS have a wide range of applications in dietary supplements and pharmaceutical drugs, while the mechanisms underlying the beneficial effects of orally administered CS remain unclear. In the present study, we investigated the possible effect of various CSs on human colonic microbiota using the in vitro human colonic microbiota model KUHIMM. The 0.3% CS added into KUHIMM together with the human fecal suspension was mostly consumed after 48 h of cultivation. KUHIMM cultures added with CS showed a substantial increase in the number of total eubacteria compared with those without CS addition, and the increases in cultures added with CH, CSC, and CSE were statistically significant (Fig. 1). These results indicate that these CSs are degraded or absorbed by the human colonic microbiota and utilized for growth. However, of note, the average molecular weights of the CSs used in this study differed (Supplementary Table S4), which could also have contributed to the degradation profiles and bacterial growth observed in this study.

Microbial composition analysis at the genus level revealed that the relative abundance of *Bacteroides* in the KUHIMM cultures tended to increase with CS addition compared with that in the control culture (Fig. 2b). Various *Bacteroides* strains possess carbohydrate-active enzymes (CAZymes) for GAG degradation and assimilation in their genomes, and are capable of catabolizing CS^13^. The results obtained in this study further support those of previous studies that *Bacteroides* strains play a major role in CS catabolism in human colonic microbiota^12, 13^.

At the species level, the relative abundance*s* of *B. ovatus* and *B. caccae* were increased by addition with any type of CS (Fig. 2e). These species can reportedly grow using CS as the sole carbon source^17^. Meanwhile, *B. plebeius*, which increased in relative abundance only with CSA, CSC, and CSE addition, has been reported to lack CS assimilation capacity because its gene cluster for CS degradation is not fully functional^17^. *B. plebeius* may have grown by utilizing the saccharides produced by other *Bacteroides* species that can degrade CS, such as *B. ovatus* and *B. caccae*. Syntrophic interactions are widespread among bacteria inhabiting the human intestine, where coexisting microorganisms enable other strains to utilize originally inaccessible polysaccharides^18^. Raghavan et al.^17^ suggested that *B. plebeius* may form a cooperative CS-utilization network with other *Bacteroides* species that can utilize CS. Our results have implications for understanding the cooperative cross-feeding of CS in the human colonic microbiota.

For the other genera, the relative abundances of *Eubacterium* and *Faecalibacterium* belonging to the phylum Firmicutes were significantly increased by CH and CSA addition (Fig. 2c,d). Several Firmicutes, including *Faecalibacterium prausnitzii*^19^, have been shown to be CS utilizers^13^. However, to our knowledge, no CS utilization by *Eubacterium* has been reported. Further research is needed to determine the causal relationship between CH addition and an increase in *Eubacterium*.

CS-degrading bacteria liberate sulfuric acid during the CS degradation and assimilation processes^20^. The sulfate released from CS becomes available to sulfate-reducing bacteria and induces their growth^21^. Shang et al.^14^ reported that the abundance of sulfate-reducing bacteria *Desulfovibrio* slightly increased after oral administration of CS and CS oligomer in mice. In the present study, the relative abundance of sulfate-reducing bacteria was very low (< 1% on average) in the fecal samples of 10 healthy individuals, and no significant increase in these bacteria was observed, even in cultures added with CS. Further studies using fecal samples containing relatively high abundances of sulfate-reducing bacteria are needed to elucidate the effects of the presence and position of sulfate groups in CS on their growth and metabolites.

Additionally, we demonstrated the effects of CS addition on the levels of SCFAs (acetate, propionate, and butyrate), which are the main products of saccharolytic fermentation of nondigestible carbohydrates in the colon. CH addition increased acetate and propionate levels, whereas CSE addition increased propionate levels (Fig. 3a,b). In the human colon, bacteria belonging to the phylum Bacteroidetes, including the *Bacteroides* genus, mainly produce acetate and propionate^22^. Since the majority of *Bacteroides* species, including *B. ovatus*, *B. caccae*, and *B. plebeius*, possess the enzymes involved in the production of these SCFAs^23^, these bacteria may have contributed to the elevated acetate and/or propionate levels. In contrast, butyrate levels were not significantly altered by CS addition (Fig. 3c). The two most dominant butyrate-producing bacteria in the human colon are *Eubacterium rectale* and *F. prausnitzii* belonging to the phylum Firmicutes^24^. Although CH and CSA addition significantly increased the relative abundance of *Eubacterium* and *Faecalibacterium* (Fig. 2c,d), they did not contribute to butyrate production. SCFAs generated in the colon play important roles in maintaining intestinal homeostasis, can be absorbed by the host, and exert various beneficial effects through a wide variety of mechanisms^25, 26^. Acetate and propionate act as natural ligands for several cell-surface G protein-coupled receptors expressed in a wide range of tissues, and they exert various biological regulatory functions, including the suppression of inflammatory responses^27, 28^, blood pressure regulation^29, 30^, and appetite suppression^31, 32^.

To further investigate the effects of CS addition, we comprehensively quantified metabolites in the supernatants of KUHIMM cultures. The trends in the effects of CH, CSC, and CSE addition on metabolite concentrations were consistent (Table 2). Addition with these CSs had a positive effect on the levels of various metabolites, mainly nitrogen-containing compounds, including amino acids and their derivatives, nucleic acid relatives, and peptides. This result suggests that CS, which contains an amino sugar (GalNAc) as a constituent monosaccharide, plays an important role as a nitrogen source as well as a carbon source for colonic bacteria. Some metabolites whose concentrations were markedly increased by CS addition have been reported to have beneficial effects on the host. Lysine is an essential amino acid for humans. It plays an important role in the human body, not only in proteinogenesis, but also in the cross-linking of collagen polypeptides^33^ and improvement of intestinal calcium absorption^34^. Ornithine is a nonproteinogenic amino acid produced in the urea cycle (also known as the ornithine cycle) that plays an important role as a hepatoprotective agent and converts excess ammonia to urea. Ornithine has been shown to promote growth hormone secretion, which eventually leads to an antifatigue effect and improves sleep and waking, skin quality, and muscle and bone development^35, 36^. Furthermore, other urea cycle intermediates, such as arginine and citrulline, also increased in concentration after CS addition (Supplementary Table S3). Oral administration of L-arginine and L-citrulline can effectively reduce blood pressure by enhancing the production of nitric oxide, a well-known vasodilator produced by the vascular endothelium^37–39^. CS addition also had a positive effect on the concentration of the bioactive peptide Ile-Pro-Pro. Ile-Pro-Pro was first identified in Japanese sour milk fermented by *Lactobacillus helveticus* and *Saccharomyces cerevisiae*^40^. It is a weak competitive angiotensin-converting enzyme inhibitor^41^, and in addition to its well-known blood pressure-lowering effect^42, 43^, anti-inflammatory and bone-protective activities have also been reported^44, 45^. These metabolites and SCFAs may be associated with some of the reported beneficial effects of orally administered CS, including anti-inflammatory^46^ and cardioprotective effects^47^. However, it should be noted that the in vitro fermentation system used in this study does not account for interactions with the intestinal tract. Whether the metabolites found in this study are physiologically associated with the beneficial effects of dietary CS requires further investigation.

In conclusion, addition with different CSs showed diverse effects on colonic microbiota and its metabolites in KUHIMM. CSA addition increased the relative abundance of *Bacteroides*, *Eubacterium*, and *Faecalibacterium* but did not increase the total number of eubacteria and SCFA levels. In contrast, CSC addition significantly increased the total number of eubacteria but did not significantly affect the genus-level bacterial composition or SCFA levels. Meanwhile, CSE addition resulted in statistically significant increases in the total number of eubacteria, relative abundance of *Bacteroides*, and SCFA levels. Furthermore, addition with CSC and CSE had a positive effect on the levels of a wide range of metabolites, including amino acids and peptides, which could have beneficial effects on the host. However, significant increases in the total number of eubacteria, relative abundance of *Bacteroides*, *Eubacterium*, and *Faecalibacterium*, and SCFA levels were also observed with addition of CH, which does not contain any sulfate group, and the trends in the effects of CH addition on metabolite concentrations were identical to those of CSC and CSE additions. This suggests that the sulfate groups of CS are not involved in the beneficial effects attributed to the metabolites of the colonic microbiota. Although further studies including human interventional clinical trials are needed, the results obtained in this study provide novel insights into the contribution of the colonic microbiota to the therapeutic effects of dietary CS.

## Methods

### Characterization of CSs

CSs (CH, CSA, CSC, and CSE) were provided by Seikagaku Co. (Tokyo, Japan). Their characteristics are listed in Supplementary Table S4. The weight-average molecular weight was determined via high-performance liquid chromatography (HPLC) (Shimadzu, Kyoto, Japan). A size exclusion column (Ultrahydrogel linear 7.8 × 300 mm; Waters, Milford, MS, USA) was used together with a refractive index detector (RID-10A; Shimadzu). The HPLC system was operated at 40 °C with 0.2 M NaCl (flow rate, 0.6 mL/min) as the mobile phase. Chromatograms of the size exclusion chromatography of CSs are shown in Supplementary Fig. S3. The weight-average molecular weights were calculated using a standard curve determined with molecular weight-defined pullulan standards (Shodex, Tokyo, Japan) (Supplementary Fig. S4). The disaccharide composition was analyzed using an HPLC apparatus equipped with a post-column fluorescent labelling system as reported previously^48^ with a slight modification. In brief, each CS was solubilized in water, and chondroitinase ABC (Seikagaku Corp.) and chondroitinase ACII (Seikagaku Corp.) were then added, followed by incubation at 37 °C for 16–18 h to completely digest the CS into their disaccharide units. The reaction solutions were ultrafiltered with a Nanosep centrifuge device with a molecular weight cutoff of 10,000 Da (Pall Corporation, Port Washington, NY, USA). The filtrates were then injected into a C22-bound silica column (SenShu Pak DOCOSIL SP400; Senshu Scientific Co., Tokyo, Japan) and eluted with a gradient of 0–140 mM sodium chloride containing 1.45 mM tetrabutylammonium monohydroxysulfate for 65 min (flow rate, 1.1 mL/min). The effluent was fluorescently labelled with 2-cyanoacetamide through a T-connector. The labelled eluates were monitored at wavelengths of 346 nm/410 nm (Ex/Em), and the area of the peak corresponding to the disaccharides was used to determine the disaccharide composition of each CS. Chromatograms of the disaccharide analysis of CSs are shown in Supplementary Fig. S5. The sulfur content was calculated as the weight of the sulfur atom (S) per weight of each CS (based on the disaccharide composition) as follows:

$${\text{Sulfur}}\,{\text{content}}\left( {\% ,{\text{ w}}/{\text{w}}} \right) = \, ({32}/{5}0{3 } \times \alpha /{1}00 \, + { 64}/{6}0{5 } \times \beta /{1}00) \, \times { 1}00,$$

where α and β are the contents (%) of unsaturated disaccharides in sodium form with one sulfate group (Δ-4,5-unsaturated hexuronic acid [ΔΗexUA] − C4-sulfated GalNAc and ΔΗexUA − C6-sulfated GalNAc) and two sulfate groups (C2-sulfated ΔΗexUA − C6-sulfated GalNAc and ΔΗexUA − C4,6-disulfated GalNAc), respectively.

### Fecal samples

Fresh fecal samples were obtained from 10 healthy Japanese volunteers, 60% of whom were female. The inclusion criteria were as follows: Japanese ancestry, no pre-existing illness (according to patient interviews), aged 20–60 years, nonsmoker, and no antibiotic treatment for at least 6 months prior to sampling. The study design was approved by the institutional ethics review board of Kobe University Hospital Clinical and Translational Research Center (research code 1902, approval date May 10, 2016), and all participants provided written informed consent before fecal sample collection. Immediately after collection, each fecal sample was stored in an anaerobic condition with a BD BBL Culture Swab (Becton, Dickinson and Company, NJ, USA) and used within 24 h. This study was conducted following the principles of the Declaration of Helsinki.

### Cultivation of fecal samples in KUHIMM

A Bio Jr.8 fermenter (ABLE, Tokyo, Japan) comprising eight parallel and independent anaerobic culturing vessels was used for fecal sample cultivation as described previously^49^. Briefly, 0.5 g of fecal samples were suspended in 2 mL of PBS buffer (nacalai tesque, Kyoto, Japan). Each vessel containing 100 mL of Gifu anaerobic medium (Nissui Pharmaceutical Co., Ltd, Tokyo, Japan) was inoculated with either 100 μL of fecal suspension alone or with 0.3% (3 g/L) of CSs and then cultivated anaerobically at 37 °C (n = 10). The culture broth was stirred at 300 rpm and continuously purged with an anaerobic gas mixture (N_2_:CO_2_ = 80:20) to maintain anaerobic conditions. After 48 h of cultivation, the culture broths were collected and used for subsequent analyses.

### Measurement of CS concentration

The supernatant of the culture broths collected at 0 and 48 h was diluted four times with water, and CS concentrations were determined by analyzing the disaccharide composition as described above. The residual CS ratio after 48 h of fermentation was calculated as follows:

$${\text{CS}}\,{\text{residual}}\,{\text{ratio }}\left( \% \right)\, = \,(\mu {\text{M}},{\text{ CS}}\,{\text{concentration}}\,{\text{in}}\,{\text{the}}\,{\text{culture}}\,{\text{broth}}\,{\text{at}}\,{48}\,{\text{h}})/(\mu {\text{M}},{\text{ CS}}\,{\text{concentration}}\,{\text{in}}\,{\text{the}}\,{\text{culture}}\,{\text{broth}}\,{\text{at}}\,0\,{\text{h}})\, \times \,{1}00.$$

### DNA extraction

Microbial genomic DNA was extracted from fecal suspensions and culture broths as described previously^16^.

### Sequencing of 16S rRNA genes

The V3–V4 region of bacterial 16S rRNA genes was amplified using the extracted DNA samples as the template, as previously described^15, 50^. Following the manufacturer’s instructions, polymerase chain reaction (PCR) was performed with an Nextera XT index adapter added to the gene sequence (Illumina Inc., San Diego, CA, USA). Amplicons were purified using AMPure XP DNA purification beads in accordance with the manufacturer’s instructions (Beckman Coulter, Brea, CA, USA). The concentration of the purified amplicons was measured using a Qubit fluorometer (Thermo Fisher Inc., Waltham, MA, USA). The amplicons were pooled at an equimolar concentration of 5 nM. The 16S rRNA genes and internal PhiX control (Illumina) were analyzed for paired-end sequencing using MiSeq (Illumina) with Reagent Kit v3 (Illumina) for 600 cycles. Pair-end reads with a Q score of 20 or higher were combined using automated CASAVA 1.8 pair-end demultiplexing FASTQ, with the FASTQ Generation in Basespace Sequence Hub (https://basespace.illumina.com/). The sequences were subjected to quality control and corrected with the DADA2 pipeline using QIIME 2 version 2022.2^51^. The OTUs were classified using the naive Bayes classifier trained on the Greengenes 13_8 99% OTU full-length sequence database. The OTUs and taxonomic metadata were used for α-diversity estimation.

### Quantification of total eubacterial growth and microbial composition analysis

Quantitative real-time PCR for the quantification of total bacterial growth and for microbial composition analysis were conducted as described previously^16^. A LightCycler 96 system (Roche, Basel, Switzerland) and the primer sets targeting all eubacteria^52, 53^ were used for the quantitative real-time PCR.

### SCFA analysis

The concentrations of acetate, propionate, and butyrate in the culture supernatants were determined via HPLC (Shimadzu), as described previously^54^.

### Metabolome analysis

The supernatants of the KUHIMM culture were filtered through a 5-kDa cut-off filter (ULTRAFREE-MC-PLHCC; Human Metabolome Technologies, Yamagata, Japan), and the filtrates were concentrated by centrifugation and resuspended in 50 μL of ultrapure water immediately before measurement. All metabolome measurements were performed using Capillary Electrophoresis Time-of-Flight Mass Spectrometry (CE-TOFMS) at a facility service at Human Metabolome Technologies Inc^55^.

### Statistical analyses

All statistical analyses in this study were performed using Prism 9 (GraphPad Software, Inc., San Diego, CA, USA). Results with *p* value < 0.05 were considered statistically significant.

## Supplementary Information

Supplementary Information 1.

Supplementary Information 2.

## Data Availability

All 16S rRNA gene sequences obtained in this study have been deposited at the MG-RAST server (http://metagenomics.anl.gov) as “Model Culture System of Human Colonic Microbiota_CSs” under accession numbers mgm4990853.3-mgm4990912.3.
