# Supplementary material for: Sulfated and non-sulfated chondroitin affect the composition and metabolism of human colonic microbiota simulated in an in vitro fermentation system
Source: Sci Rep. 2023 Jul 29;13:12313. doi: 10.1038/s41598-023-38849-5 (PMC10387111; doi:10.1038/s41598-023-38849-5)
Supplement: Supplementary file 1 — Supplementary Information 1. [file 41598_2023_38849_MOESM1_ESM.pdf]

Supplementary Information

Scientific Reports

Sulfated and non-sulfated chondroitin affect the composition and metabolism of human colonic microbiota simulated in an *in vitro* fermentation system

Kentaro Inokuma,[a] Daisuke Sasaki,[a] Kaoru Kurata,[b] Megumi Ichikawa,[b] Yuya Otsuka,[b] Akihiko Kondo,[a,c,*]

[a] Graduate School of Science, Technology and Innovation, Kobe University, 1-1 Rokkodai-cho, Nada-ku, Kobe 657-8501, Japan

[b] Glycoscience, Central Research Laboratory, Seikagaku Corporation, 3-1253, Tateno, Higashiyamato, Tokyo 207-0021, Japan.

[c] Biomass Engineering Program, RIKEN, 1-7-22 Suehiro-cho, Tsurumi-ku, Yokohama, Kanagawa 230-0045, Japan

* Corresponding author:

Akihiko Kondo

Telephone: +81-78-803-6196, Fax: +81-78-803-6196

E-mail: akondo@kobe-u.ac.jp

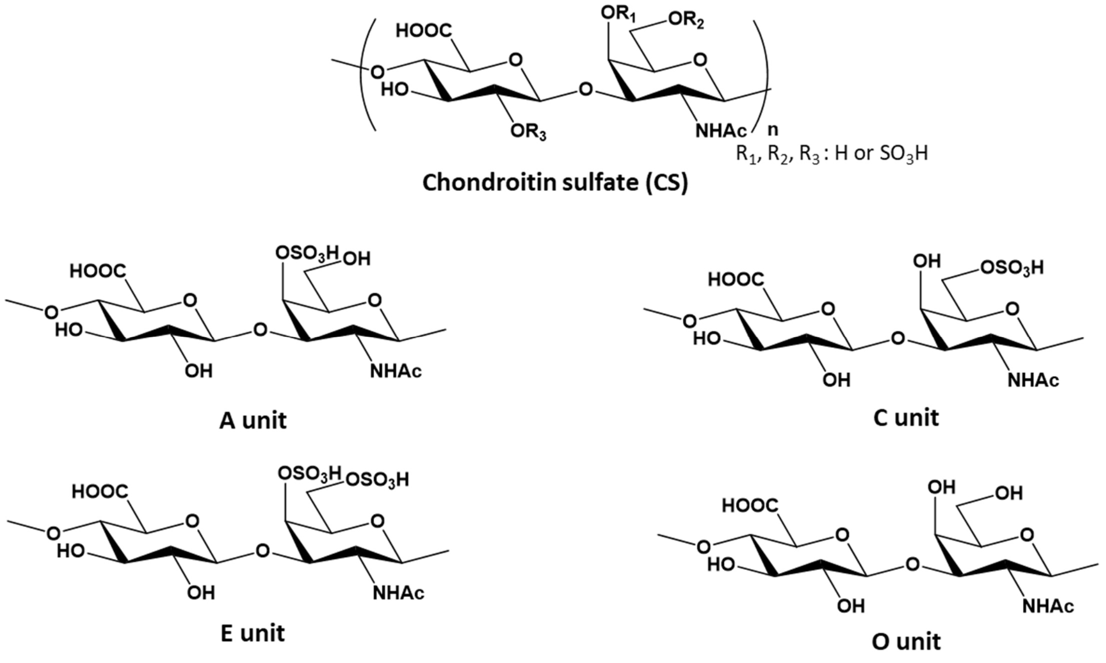

Figure S1 Chemical structures of CS and CS disaccharide units.

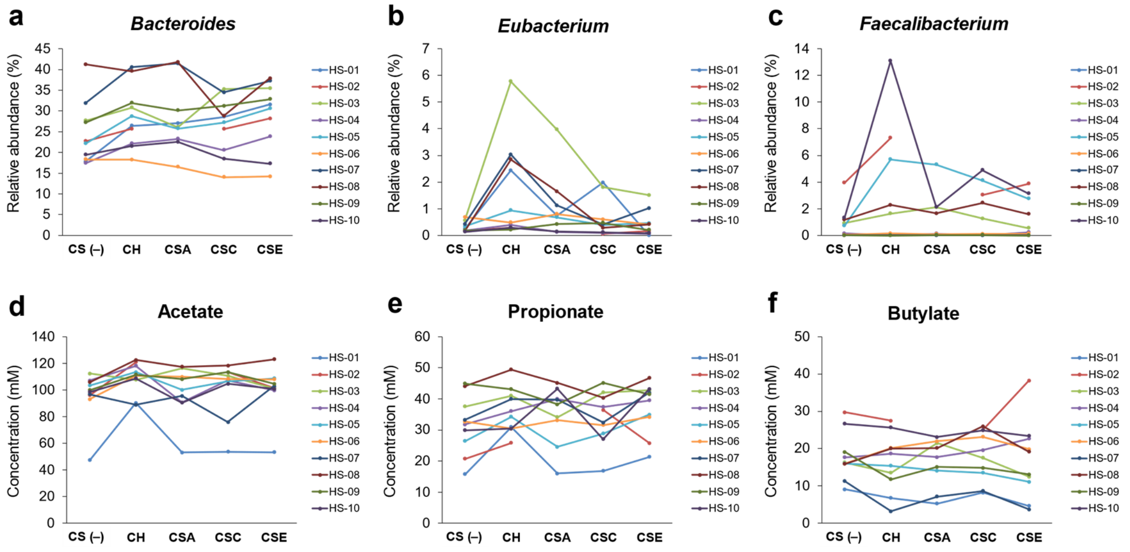

Figure S2 Changes in the relative abundances of the genus *Bacteroides* (**a**), *Eubacterium* (**b**), and *Faecalibacterium* (**c**) and the concentrations of acetate (**d**), propionate (**e**), and butyrate (**f**) after 48 h of cultivation in KUHIMM inoculated with each fecal sample.

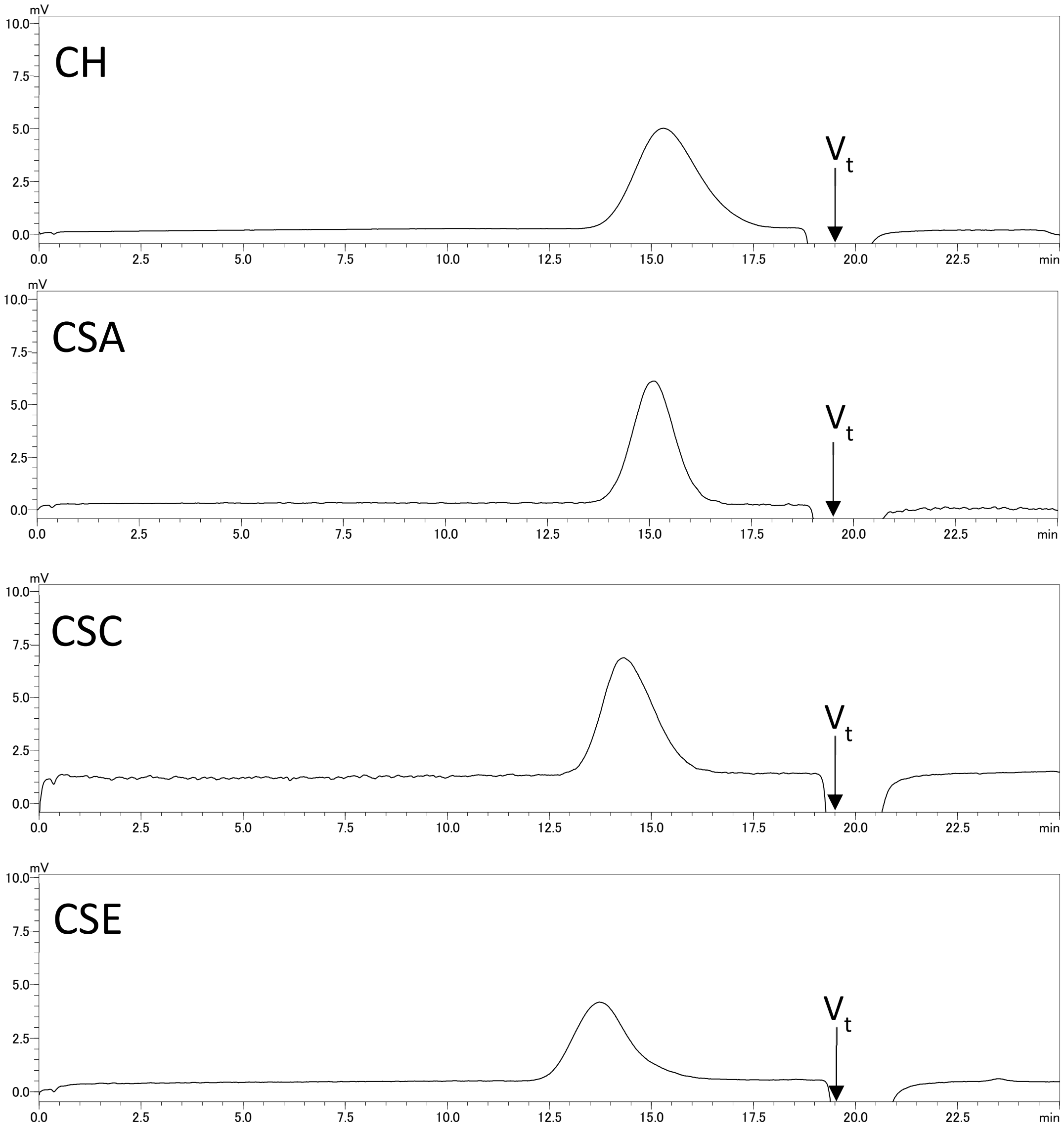

Figure S3 Chromatograms of size exclusion chromatography to determine the weight-average molecular weight of CSs. Column; Ultrahydrogel Linear, 7.8 mm (i.d.) x 300 mm (Waters Corporation). Flow rate; 0.6 mL/min. Solvent; 0.2 M NaCl. Detector; Refractive index detector (Shimadzu Corporation). Black arrows indicate the total retention volume (V_t).

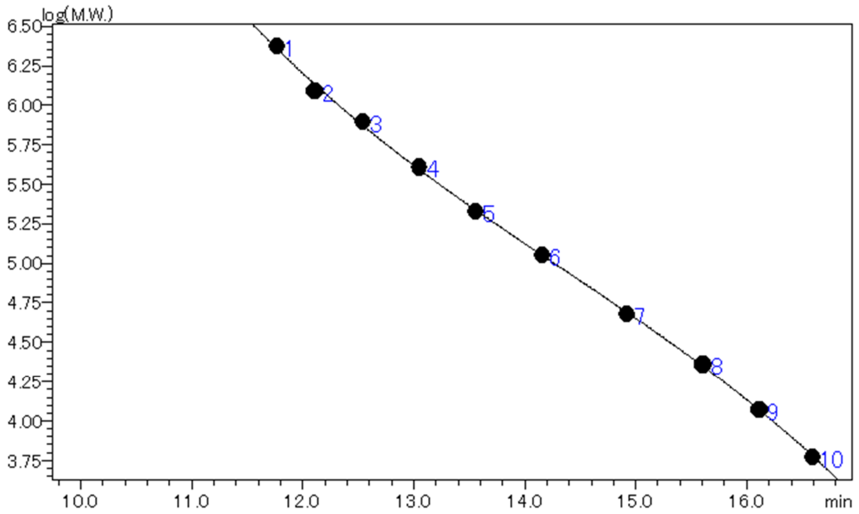

No.	Standard name	Weight average molecular weight	Retention time (min)
1	Pullulan STD P-2500	2350000	11.775
2	Pullulan STD P-1300	1220000	12.112
3	Pullulan STD P-800	788000	12.541
4	Pullulan STD P-400	404000	13.049
5	Pullulan STD P-200	212000	13.564
6	Pullulan STD P-100	112000	14.160
7	Pullulan STD P-50	47300	14.926
8	Pullulan STD P-20	22800	15.609
9	Pullulan STD P-10	11800	16.117
10	Pullulan STD P-5	5900	16.599

Figure S4 Standard curve with molecular weight-defined pullulan standards applied for calculation of the weight-average molecular weight of CSs. The HPLC condition was the same as described in Figure S3.

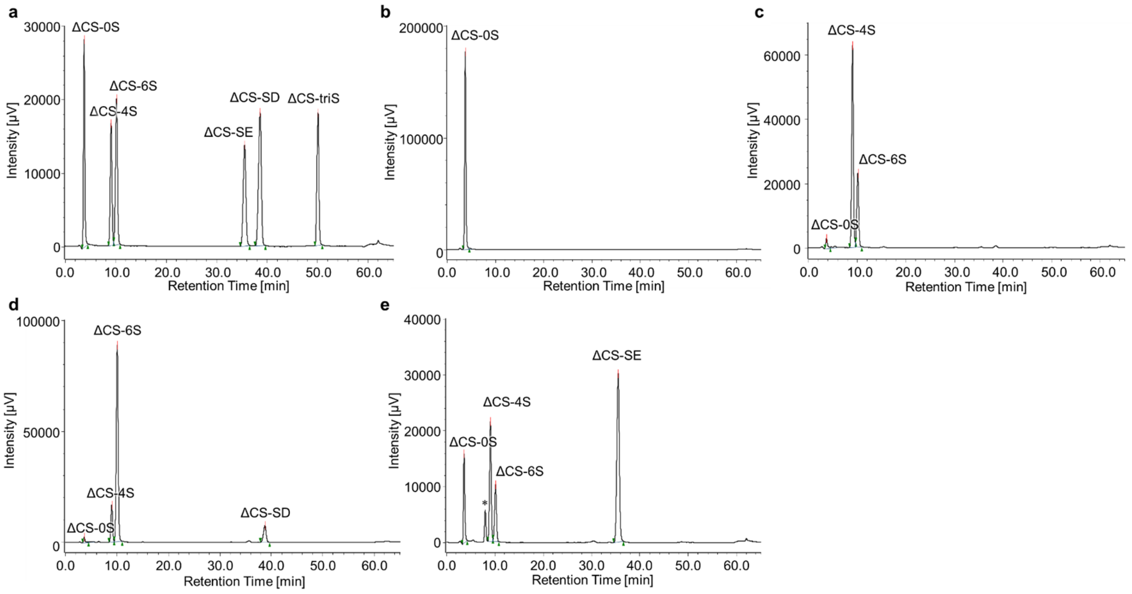

Figure S5 Chromatograms of disaccharide analysis of unsaturated disaccharide standards (**a**), CH (**b**), CSA (**c**), CSC (**d**), and CSE (**e**). Abbreviations of unsaturated disaccharides are as follows: ΔCS-0S, ΔHexUA–GalNAc; ΔCS-4S, ΔHexUA–GalNAc (4-*O*-sulfate); ΔCS-6S, ΔHexUA–GalNAc (6-*O*-sulfate); ΔCS-diS$_{D}$, ΔHexUA (2-*O*-sulfate)–GalNAc (6-*O*-sulfate); ΔCS-diS$_{E}$, ΔHexUA–GalNAc (4,6-*O*-disulfates); ΔCS-triS, ΔHexUA (2-*O*-sulfate)–GalNAc (4,6-*O*-disulfates). The asterisk indicates a low content trisaccharide observed in CSE from squid cartilage. Column; SenShu Pak DOCOSIL SP400, 4.6 mm (i.d.) x 150 mm (Senshu Scientific Co., Ltd.). Flow rate; 1.1 mL/min. Solvent A; 1.45 mM tetrabutylammonium monohydroxysulfate. Solvent B; 140 mM sodium chloride containing 1.45 mM tetrabutylammonium monohydroxysulfate. Gradient; 0%B to 100%B in 65 min. Detector; Fluorescent detector (JASCO Corporation).

Table S1 Concentrations of each type of CS in the culture broth at 0 and 48 h

Fecal samples	Concentration (mg/L)[a]							
	CH		CSA		CSC		CSE	
	0 h	48 h	0 h	48 h	0 h	48 h	0 h	48 h
HS-01	2842.6	79.8	2831.6	117.2	3169.4	133.7	2414.3	213.8
HS-02	2488.4	41.4	2863.8	3068.9	2986.7	40.2	2522.9	36.3
HS-03	2789.0	40.7	2576.3	39.6	3098.3	43.3	2533.6	46.7
HS-04	2577.4	35.5	2980.7	49.7	2745.8	41.3	2476.8	44.4
HS-05	2867.9	37.6	3033.1	39.7	3059.3	37.8	2657.7	40.6
HS-06	2801.6	34.9	2902.6	36.4	2773.6	36.2	2350.6	42.1
HS-07	2572.3	44.4	2886.1	44.3	2989.4	44.1	2616.1	445.2
HS-08	2844.1	33.1	2907.5	37.6	3172.0	31.7	2438.4	36.0
HS-09	2709.4	40.3	2737.9	38.2	2899.8	40.3	2398.3	46.9
HS-10	2929.0	77.4	2586.6	40.4	3112.8	285.7	2288.6	42.6

[a]The concentration of CSs in the samples was found by summing the concentrations of unsaturated disaccharides determined by HPLC analysis described in Figure S5.

Table S2 Summary of 16S rRNA gene sequencing data and α-diversity values

	High-quality leads	Observed OTUs		Chao1		Shannon index		Simpson index	
		mean ± SD	*p*-values	mean ± SD	*p*-values	mean ± SD	*p*-values	mean ± SD	*p*-values
FEC	112,775 ± 34,261	263 ± 103	-	273 ± 108	-	5.83 ± 0.57	-	0.96 ± 0.01	-
CS(–)	135,407 ± 11,906	167 ± 40*	0.029[a]	181 ± 43	0.052[a]	5.02 ± 0.39**	0.002[a]	0.93 ± 0.02**	0.003[a]
CH	133,509 ± 17,768	152 ± 47	0.388[b]	169 ± 50	0.535[b]	4.99 ± 0.28	0.905[b]	0.94 ± 0.01	0.968[b]
CSA	129,411 ± 16,538	145 ± 38	0.127[b]	162 ± 41	0.315[b]	5.00 ± 0.30	0.631[b]	0.93 ± 0.02	0.739[b]
CSC	143,248 ± 23,505	153 ± 38	0.325[b]	176 ± 39	0.684[b]	4.96 ± 0.32	0.912[b]	0.93 ± 0.02	>0.999[b]
CSE	117,546 ± 39,224	147 ± 39	0.211[b]	163 ± 38	0.287[b]	4.89 ± 0.35	0.604[b]	0.93 ± 0.02	0.661[b]

Ten human fecal samples (FEC), the corresponding cultures without chondroitin sulfate (CS(–)), and the corresponding cultures with each CS (nonsulfated chondroitin [CH] and CSs sulfated C-4, C-6, and both positions of the *N*-acetylgalactosamine [GalNAc] residues [CSA, CSC, and CSE], respectively) were sampled after 48 h of cultivation. Values are presented as mean ± standard deviation (SD). Significant differences in the α-diversity values were detected using Mann–Whitney *U*-test (* $p < 0.05$ and ** $p < 0.01$). OTU, operational taxonomic units.

[a]These are the *p*-values vs. FEC group.

[b]These are the *p*-values vs. CS(–) group.

Table S4 Characteristics of each CS used in this study

CS family	Sulfur content (%, w/w)	Disaccharide composition (mol %)						Weight average molecular weight (kDa)	Source
		CS-0S	CS-4S	CS-6S	CS-diS$_E$	CS-diS$_D$	CS-triS		
CH	0.0	100.0	0.0	0.0	0.0	0.0	0.0	37	Recombinant *E. coli*[1]
CSA	6.2	2.3	74.6	23.1	0.0	0.0	0.0	46	Whale cartilage
CSC	6.6	1.2	16.9	73.5	0.0	8.3	0.0	97	Shark cartilage
CSE	8.0	9.8	26.3	10.4	53.6	0.0	0.0	203	Squid cartilage

Disaccharide composition of each CS was determined by disaccharide analysis. Abbreviations of CS disaccharide units are as follows: CS-0S, GlcUA–GalNAc; CS-4S, GlcUA–GalNAc (4-*O*-sulfate); CS-6S, GlcUA–GalNAc (6-*O*-sulfate); CS-diS$_D$, GlcUA (2-*O*-sulfate)–GalNAc (6-*O*-sulfate); CS-diS$_E$, GlcUA–GalNAc (4,6-*O*-disulfates); CS-triS, GlcUA (2-*O*-sulfate)–GalNAc (4,6-*O*-disulfates).

Reference in the Supplementary Information

1. Doherty, D. H., Weaver, C. A., Miyamoto, K., Minamisawa, T. Compositions and methods for bacterial and genetically modified microorganism production of chondroitin. US Patent 9, 175, 293 B2 (2017).
